# Dr. Mrs. Dhanalakshmi De Sousa 1938–2005: A Tribute

**Published:** 2006

**Authors:** Avinash De Sousa

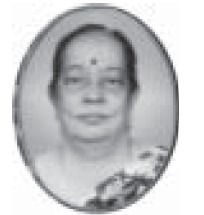



It is always tough when one has to write a tribute to one's mother and tougher still when the same has to be written after her demise and when we remember her most. Yet I undertake this onerous task. Many people know her as the past Professor and Head of the Department of Psychiatry at Lokmanya Tilak Municipal Medical College and General Hospital, but it is the personal side of her personality that I wish to highlight here.

Born on 3^rd^ October 1938 and brought up in a traditional South Indian family, she lost her father at an early age when she was just 8 years old. Yet she struggled and studied hard, her efforts earning her an admission for the medical graduation course at Seth G.S. Medical College, Mumbai. She joined the Department of Psychiatry at K.E.M. Hospital in 1965 under Dr. V.N. Bagadia and said it was chance that led her to do so. Later she told me it was her destiny, as she had to meet her love and husband Dr. Alan De Sousa there.

She was instrumental in starting the Child Guidance Clinic at Nair Hospital that has today risen to be one of the best in the city. She joined Sion Hospital in 1971 as a Honorary Professor and had an avid interest in teaching both post graduates and under graduate students. She worked there till 1989 when she retired as the Prof and Head of the Department. Her retirement was premature for which I take the blame. She always knew what her priorities were and gave me all the best she could. Once when I told her that she was too busy and did not have time for me, it was the very next day that she submitted her resignation from the Department. It was a courageous decision, one for which I hope I fulfill all her dreams.

She had a special interest in child psychiatry, substance abuse and psychiatric problems in women.

She was always humane and went out of her way to help the poor and the needy. She always taught me to help everyone in whichever way you could, and spread your knowledge, for when you spread knowledge it does not divide, rather it multiplies. She has taught me that in all you do and earn, always give something back to society in whatever way you can. She had a keen interest in research and had published a number of research papers, most of which were done in an era when journals were scarce and computers unavailable.

She believed that if psychiatry must be learnt, it must be learnt with love and not just as a subject. For the same an award was instituted in her name for undergraduate students in psychiatry that shall bear her name forever.

She was very famous for her Christmas parties, as many people tell me, where they remember digging into various types of food of varying cuisines all cooked and picked individually by her.

She has been my guiding force and a force for Dad. We shall always miss her as she is irreplaceable.

I end with these words that are apt for her –

God looked around his Garden and found an empty place.

He then looked down upon his earth and saw your loving face.

He put his arms around you and lifted you to rest.

His Garden must be beautiful, he always takes the best.

You've left us precious memories, your love will be our guide,

You live on through your children, you're always by our side.

It broke our hearts to lose you, but you did not go alone.

For part of us went with you the day God called you home.

## Dr. Avinash De Sousa

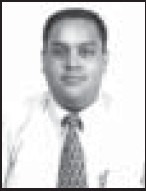



A Consultant Psychiatrist by profession but has a keen interest in holistic medicine and research of various kinds. He has an active interest in Child and Adolescent Psychiatry, works in these areas with numerous schools and colleges as a consultant school psychiatrist and counsellor. He also has attachments to special schools and autism centers in the same field. His second area of interest is Substance Abuse Pharmacotherapy with his area of research being Disulfiram. He has published two books in the field with over 20 publications to his name. He has a firm belief that mental health needs teamwork and a team effort to achieve its goal. avinashdes999@yahoo.co.uk

